# Sex and age affect depot expression of Ca^2+^ channels in rat white fat adipocytes

**DOI:** 10.1530/JME-23-0108

**Published:** 2024-02-28

**Authors:** Yan Meng, Maria Toledo-Rodriguez, Olena Fedorenko, Paul A Smith

**Affiliations:** 1 School of Life Sciences, University of Nottingham, Queen’s Medical Centre, Nottingham, UK

**Keywords:** sex, mammogenesis, fat, transcriptomics, adipogenesis

## Abstract

White adipose tissue (WAT) requires extracellular Ca^2+^ influx for lipolysis, differentiation, and expansion. This partly occurs via plasma membrane Ca^2+^ voltage-dependent channels (CaVs). However, WFA exists in different depots whose function varies with age, sex, and location. To explore whether their CaV expression profiles also differ we used RNAseq and qPCR on gonadal, mesenteric, retroperitoneal, and inguinal subcutaneous fat depots from rats of different ages and sex. CaV expression was found dependent on age, sex, and WFA location. In the gonadal depots of both sexes a significantly lower expression of CaV1.2 and CaV1.3 was seen for adults compared to pre-pubescent juveniles. A lower level of expression was also seen for CaV3.1 in adult male but not female gonadal WFA, the latter of whose expression remained unchanged with age. Relatively little expression of CaV3.2 and 3.2 was observed. In post-pubescent inguinal subcutaneous fat, where the third and fourth mammary glands are located, CaV3.1 was decreased in males but increased in females – thus suggesting that this channel is associated with mammogenesis; however, no difference in intracellular Ca^2+^ levels or adipocyte size were noted. For all adult depots, CaV3.1 expression was larger in females than males – a difference not seen in pre-pubescent rats. These observations are consistent with the changes of CaV3.1 expression seen in 3T3-L1 cell differentiation and the ability of selective CaV3.1 antagonists to inhibit adipogensis. Our results show that changes in CaV expression patterns occur in fat depots related to sexual dimorphism: reproductive tracts and mammogenesis.

## Introduction

Obesity and its clinical management is a worldwide problem. Obesity is associated with increase in morbidity and mortality rates through predominantly cardiovascular and metabolic dysfunction, as well as an increased risk of cancer. In particular, it is well established that obesity is associated with both an increased risk of post-menopausal breast cancer as well at its reoccurrence, where weight loss reduces this risk (Jiralerspong & Goodwin 2016, Kothari *et al.* 2020). White fat adipose (WFA) is a major component of mammary gland tissue and undergoes various morphological and functional changes with puberty, pregnancy, lactation, involution, and menopause (Kothari *et al.* 2020, Colleluori *et al.* 2021). Indeed, the intimate relationships between WFA and mammary gland function are suggested as an ideal microenvironment for the initiation and maintenance of breast cancer (Kothari *et al.* 2020, Colleluori *et al.* 2021).

Obesity is due to increased WFA mass through hypertrophy and hyperplasia of white fat adipocytes. Adipocytes primarily act as an energy reservoir to store excess energy as triglycerides via lipogenesis during times of excess and release energy, as lipids via lipolysis, in times of demand (Arner *et al.* 2011). Many functions of WFA are regulated by Ca^2+^; this makes Ca^2+^ metabolism a potential therapeutic target to regulate obesity (Fedorenko *et al.* 2020). For example, lipolysis in WFA is promoted by extracellular Ca^2+^ influx though voltage-gated calcium channels (CaVs) (Izawa *et al.* 1983, Fedorenko *et al.* 2020, Akaniro-Ejim & Smith 2021). CaV-mediated Ca^2+^ influx also helps explain why changes in extracellular Ca^2+^ can modulate proliferation, differentiation, and expansion of adipocytes – processes that underlie the hypertrophy and hyperplasia of adipose tissue associated with obesity (Oguri *et al.* 2010, Sun *et al.* 2012, Zhang *et al.* 2018). Interestingly, these processes are differentially regulated between males and females (Demerath *et al.* 2007, Chang *et al.* 2018, Schorr *et al.* 2018).

Consequently, given the multiple effects of CaVs activity in WFA, CaVs may be a novel target to effect fat disposition – a notion already supported by the ability of CaV antagonists to decrease FFA levels *in vivo* (Hvarfner *et al.* 1988, Cignarella 1994) as well as impede weight gain in mice (Uebele *et al.* 2009).

The properties of CaVs are extensively documented (Catterall 2011, Zamponi *et al.* 2015). CaVs are heteromeric structures organised around a membrane-intrinsic alpha_1_ subunit; alpha_1_ forms the ion channel pore, imparts voltage sensitivity and supports drug binding (Zamponi *et al.* 2015). On the other hand, the intracellular accessory subunits, beta-, gamma-, and alpha_2_-delta, are membrane-extrinsic proteins that modify alpha_1_ subunit expression and function (Dolphin 2016). The CaV alpha_1_ subunit is encoded by 10 genes, which are grouped into three classes: L-type, CaV1.1-CaV1.4; P-, Q-, and N-type, CaV2.1-2.3; T-type, CaV3.1-3.3. Each class has its own unique properties of voltage sensitivity, inactivation, biophysics, pharmacology, and regulation – properties further diversified by tissue-dependent splice variation, phosphorylation, and subunit combination (Catterall 2011, Zamponi *et al.* 2015, Dolphin 2016).

Previous work from our laboratory has demonstrated the presence of mRNA transcripts and protein expression for the alpha_1_ subunit for L-type CaVs CaV1.2 (*Cacna1c*) and CaV1.3 (*Cacna1d*), as well their accessory subunits, in adipocytes isolated from rat epididymal fat pads (Fedorenko *et al.* 2020). Mainly because of size and accessibly, epididymal fat is the foremost established model to explore visceral WFA physiology (Chusyd *et al.* 2016). However, other visceral as well as subcutaneous fat depots are recognised in the aetiology of metabolic disease (Arner *et al.* 2011, Chusyd *et al.* 2016). Moreover, epididymal fat is exclusively male in origin, but since the aetiology of human metabolic disorders and fat pad patterning itself are sex dependent (Chusyd *et al.* 2016, Valencak *et al.* 2017), an exploration of sexual dimorphism in WFA physiology and CaV expression is needed. For example, western blots of heart tissue demonstrated changes in CaV expression with puberty and sexual maturation (Sims *et al.* 2008). To date, the expression profile of CaVs in murine WFA depots and how these vary with sex and puberty are unknown. Electrophysiological approaches conventionally provide unequivocal functional characterisation of CaVs; however, its application to primary fat cells is technically difficult (Bentley *et al.* 2014, Fedorenko *et al.* 2020, Akaniro-Ejim & Smith 2021). Secondly, given the common mesenchymal origin of different fat depots, modification of the function and development of a specific fat depot via conditional transgenic knockout becomes problematic. Thirdly, transgenic approaches for these channel types are confounded by compensatory mechanisms (Namkung *et al.* 2001, Uebele *et al.* 2009, Wang *et al.* 2016). Consequently, we have used transcriptomics as a pragmatic method to explore CaV expression in WFA.

Our primary aim was to use molecular biological approaches to explore how the normative expression levels of CaVs vary with depot, age, and sex in WFA deposits from rats under *ad libitum* feeding conditions. The secondary aim was to determine if CaV activity affects adipogenesis of preadipocytes.

## Materials and methods

### Isolation and preparation of adipocytes

Animal care and experimental procedures were locally approved (ASPA 000187, University of Nottingham) and performed in accordance with the UK Home Office Animals (Scientific Procedures) Act (1986). Rats were obtained from Charles River (RRID: RGD_2308816), kept group housed with a 12-h light–12-h darkness cycle and fed *ad libitum*. Rats were killed by cervical dislocation. Fat depots were excised from pre-eostropausal virgin adult (age> 140 days postpartum but < 270 days postpartum; 280–300 g) and juvenile (age 14–30 days postpartum) Wistar rats. As a source for subcutaneous fat, we used the inguinal depots (IWAT). For visceral fat we used three different sources: retroperitoneal, mesenteric, and gonadal depots; where for the latter, epididymal fat was used from males and periovarian fat from females. Primary white fat adipocytes were prepared from depot explants as previously described (Bentley *et al.* 2014). Dissection and isolation of adipocytes was performed in a Hanks’ buffer solution supplemented with 5 mM glucose and 0.5% wt/vol BSA (Sigma A3058). After excision, depots were washed in Hanks’ and visible blood vessels expurgated. Depots were cut into ~5 mm pieces and added to a 25 mL Nalgene Erlenmeyer flask which contained 6 mL of Hanks’ with 1 mg mL^−1^ type II collagenase (Sigma C6885) and minced for 1 min. Adipocytes were liberated by a 30–50-min collagenase digestion with mild agitation at 37°C. Digestion progress was indicated by the appearance of the buoyant band of adipocytes. Digestion was halted by dilution and cooling via the addition of 20 mL of Hanks’ at 20–22°C. The digest was then filtered through a 250 µM nylon mesh (Normesh limited, Oldham, UK) and the filtrate collected in an inverted 50 mL syringe. Adipocytes separated from the buffer by flotation to form a buoyant layer, whereas cell debris and other cell types precipitated out. After 5 min the infranatant and cell debris were removed and the process of separation by flotation repeated twice more by further additions of 20 mL of buffer and subsequent drainage. After the final drain, the adipocytes were suspended in 2 mL Hanks’ solution, spun down at 1000 ***g*** for 10 min to obtain packed cells for subsequent molecular biology.

Differentiated 3T3-L1 cells, an established model of mouse IWAT were prepared and validated as detailed elsewhere (Bentley *et al.* 2014). Briefly, 3T3-L1 pre-adipocytes (passage number less than 17) were differentiated into adipocytes by exposure to DMEM media plus 10% fetal bovine serum (FBS) supplemented with 1 µg/mL insulin, 0.5 mM IBMX, 0.25 µM dexamethasone, and 2 µM rosiglitazone. They were subsequently maintained in DMEM plus 10% FBS alone. Differentiation was confirmed by Oil Red O staining. Experiments were carried out on day 8.

### RNA extraction

Total RNA was extracted from either freshly isolated adipocytes, or those previously frozen at −80ºC. This was performed with a RNAeasy Lipid Tissue kit, which included QIAzol Lysis Reagent, in accordance with the manufacturer’s instructions (Qiagen).

### RNA sequencing

RNA quantity was determined with the Qubit RNA BR Assay Kit and Qubit fluorometer (ThermoFisher, RRID: SCR_018095). RNA integrity was assessed with the Agilent TapeStation 4200 and Agilent RNA ScreenTape Assay Kit (Agilent). mRNA was then purified from 1 µg of total RNA with the NEBNext Poly(A) mRNA Magnetic Isolation Module (E7490, New England Biolabs, Hitchin, Hertfordshire, UK). Indexed sequencing libraries were prepared with the NEBNext Ultra II Directional RNA Library Preparation Kit for Illumina (New England Biolabs) and NEBNext Multiplex Oligos for Illumina (96 Unique Dual Index Primer Pairs, New England Biolabs). Constructed Libraries were quantified using the Qubit Fluorimeter (ThermoFisher Scientific, RRID: SCR_018095) and the Qubit dsDNA HS Kit (ThermoFisher Scientific) and their fragment-length distributions analysed with the Agilent TapeStation 4200 and the Agilent High Sensitivity D1000 ScreenTape Assay (Agilent). The libraries were then pooled in equimolar amounts and a final library quantification performed using the KAPA Library Quantification Kit for Illumina (Roche). The library pool was sequenced on an Illumina NextSeq 500 (RRID: SCR_014983; Illumina), to generate over 100 million pairs of 75-bp paired-end reads per sample. Trimming and analysis of obtained data were performed with Galaxy (RRID: SCR_006281) and Integrated Genome Viewer software (RRID: SCR_011793).

The homogeneity and purity of our isolated adipocyte preparations were tested by the absence of RNA for the specific markers of other cell types present in WFA: sympathetic neurons, synaptic vesicle monoamine transporter (VMAT), neuronal nuclei antigen (NeuN); parasympathetic neurons; choline *O*-acetyltransferase (Chat), neuronal Nuclei antigen (NeuN); astrocytes, glial fibrillary acidic protein (GFAP); endothelia, endothelial cell adhesion molecule 1 (CD31); smooth muscle, smooth muscle protein 22-alpha (SM22) and the L-type voltage-gated calcium channel alpha_1_ subunit (CaV1.1, Cacna1s); and for macrophages transmembrane immune signaling adaptor (Tyrobp) and adhesion G protein-coupled receptor E1 (Adgre1). In the RNA-seq, fragments per kilobase of transcript per million mapped reads (FPKM) for all markers but macrophage were zero, with geometric mean FPKMs of 14 for Tyrobp and 1.2 for Adgre1, which is indicative of macrophage contamination (Ying *et al.* 2017). Reads of specific markers of white fat adipocytes – adiponectin (ADIPOQ), leptin (LEP), and S100 calcium-binding protein B (S100B) – had respective geometric mean FPKMs of 1415, 421, and 540.

### Quantitative polymerase chain reaction

The concentration of isolated RNA was determined with a NanoDrop Spectrophotometer (Thermofisher) and its quality checked via an Agilent bioanalyzer (Agilent). RNA samples were treated with DNase I (Thermofisher) to remove genomic DNA, and cDNA synthesis was performed using 1 µg of total RNA with SuperScript™ III Reverse Transcriptase (10 units/µL RNA, Thermofisher).

Standard PCRs were performed in a total volume of 25 µL using DreamTaq PCR MasterMix (Thermofisher), with 2 µl of cDNA product (from a 20 µL RT reaction) and 1.25 µM of each specific primer pair (Table 1). PCR was performed at 95°C for 10 min, followed by 95°C for 15 sec then 40 cycles at the specific primer annealing temperature, and 72°C for 35 s. The last cycle was followed by a final extension step at 72°C for 10 min. PCR products were size fractionated on 1% (w/v) agarose gels stained with SYBR™safe (Thermofisher) and imaged under UV light with GeneSnap software (Syngene, Cambridge, Cambridgeshire, UK). Generated cDNAs were sequenced to identify the PCR product which confirmed the channel type. Quantitative polymerase chain reaction (qPCRs) was used to determine gene expression level. qPCRs were performed in triplicate with SYBR® Green JumpStart TM Taq ReadyMix^TM^ (Sigma) master mix, and detection was performed using the Rotor-Gene 6000 cycler (Corbett Research, Cambridge, Cambridgeshire, UK). qPCR was performed at 95°C for 10 min, followed by 40 cycles of denaturation at 95ºC for 15 s, 30 s at the specific primer annealing temperature, and elongation at 72ºC for 40 s. For reference, three housekeeping genes were used: TATA-box, GAPDH, and PGK-1.

**Table 1:** PCR/qPCR primers used for amplification of voltage-gated Ca2+ channel and housekeeping genes.

Gene	Forward primer	Reverse primer	Annealing temperature (^o^C)
TATA-box	CAGCCTTCCACCTTATGCTC	TGCTGCTGTCTTTGTTGCTC	63
GAPDH	GGCAAGTTCAATGGCACAGT	TGGTGAAGACGCCAGTAGACTC	63
Pgk_1	TAGTGGCTGAGATGTGGCACAG	GCTCACTTCCTTTCTCAGGCAG	63
Cacnca1a	CGTCATCAAACCGGGTACA	GTCGAAGTTGGTGGGAGGAG	62
Cacnca1b	CTCCAGCGTAAACTCACCG	TTGTCCCTATCACGATGCC	62
Cacna1c	CGCATTGTCAATGACACGATC	CGGCAGAAAGAGCCCTTGT	58
Cacna1d	TTGGTACGGACGGCTCTCA	CCCCACGGTTACCTCATCAT	58
Cacnca1e	TGTGTGGCCATCGTTCATCA	TCGGAAGTTGCCCAAACGT	62
Cacnca1f	AGCACAAGACCGTAGTGGTG	ATACCCCCAATGCCACACAG	58
Cacnca1g	TACTTTGGCCGGGGAATC	TCTCCCACACACTGATGACC	58
Cacnca1h	GTGAGTGTACCCGTGAGGACAA	TTTCCTGTGCTGTAGGTGGG	62
Cacnca1i	CGGAAAGCTGGTCTGCAAT	GAACTGAGCTGTGAGCACGAA	62
Cacna1s	GCAGTGCGTGTTTGTTGCTA	ACTCTATCTGCGTGGGGTCT	58

The resultant mean threshold cycle (Ct) values were used for reference gene normalisation and gene expression analyses. Relative quantification was performed by the method of Pfaffl (Pfaffl 2001).

### Effect of CaV blockers on adipogenesis

TTA-A2, IC_50_ ~5 µM (Kraus *et al.* 2010) and Calciseptine IC_50_ ~10 nM (De Weille *et al.* 1991) selective blockers of CaV3.x and CaV1.x, respectively, as well as mibefradil, which has 12–13 greater potency on CaV3.x, IC_50_ ~1 µM than CaV1.x (Martin *et al.* 2000) (Alomone, Jerusalem, Israel), were applied to 3T3-L1 cells prior to and post differentiation with each media exchange. On the eighth day, cell proliferation was determined by nuclear density and differentiation by lipid content. Nuclei were stained with Hoechst 33342 (ThermoFisher) at 10 µg mL^-1^ in Hanks’ for 30 min. After twice washing in PBS, lipid was stained by 10 µg mL^−1^ Nile Red (NR) (ThermoFisher) in Hanks’ for 10 min. Dye content was detected with a SpectraMax M2 microplate spectrophotometer (Molecular Devices) with 350 nm excitation/490 nm emission for Hoechst 33342 and 510 nm excitation/590 nm emission for NR. After background correction, the NR to Hoechst fluorescence ratio was taken as an index of adipogenesis. Images were captured with a Zeiss ERc 5rs Axiocam attached to an Zeiss Axio with a 20× objective and filter sets as indicated earlier. Images were acquired with Zeiss Zen software and analysed with Digimizer (MedCalc Software Ltd).

### Statistical analysis

Statistical analyses were performed using GraphPad PRISM Ver. 10.1 (RRID: SCR_002798). Distributions were checked for normality with the D'Agostino and Pearson omnibus test. Inferential tests are given in the text. Data are shown as scatter plots with the mean ± s.d. or as box plots with the median, 25–75% interquartile and 5–95% ranges. Data are given as mean ± s.d. or median with 5–95% CI to three significant figures, where *n* is the number of different animal preparations. Unless stated otherwise statistically significance was assigned when *P* < 0.05, and statistically significant values, in graphics, are flagged as follows: *P < 0.05, **P < 0.01, ***P < 0.001, and ****P < 0.0001.

## Results

**Figure 1:**
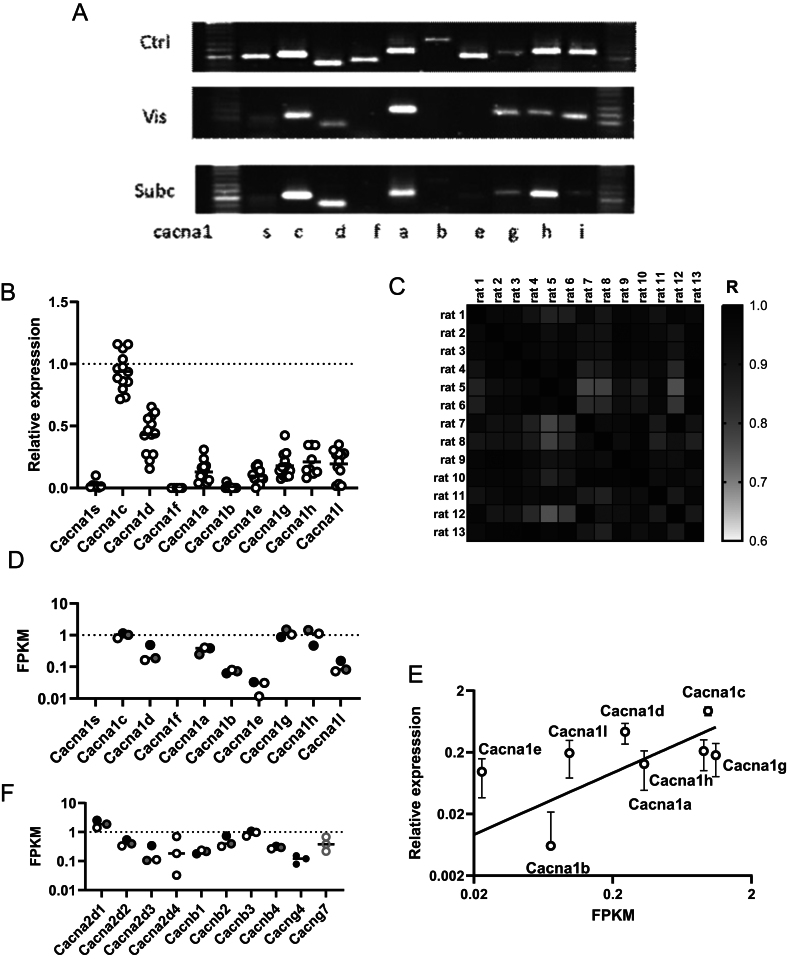
Expression of *Cacna1* genes in isolated adipocytes from purified visceral epididymal adipocytes of adult male rat. (A) Representative RT-PCR products of voltage-dependent L-type calcium channels alpha_1_ subunits for brain (Ctrl), epididymal fat (Vis) and inguinal subcutaneous fat (Subc). Ctrl, rat brain; Vis, epididymal; Subc, inguinal subcutaneous fat; *Cacna1s* (CaV1.1); *Cacna1c* (CaV1.2); *Cacna1d* (CaV1.3); *Cacna1f* (CaV1.4); *Cacna1a* (CaV2.1); *Cacna1b* (CaV2.2); *Cacna1e* (CaV2.3); *Cacna1g* (CaV3.1); *Cacna1h* (CaV3.2); *Cacna1i* (CaV3.3). (B) Relative expression of CaV alpha_1_ subunits in male epididymal visceral fat as determined by qPCR normalized to mRNA expression of CaV1.2; (*n* = 5–13). Ctrl, rat brain; Vis, epididymal; Subc, inguinal subcutaneous fat; *Cacna1s* (CaV1.1); *Cacna1c* (CaV1.2); *Cacna1d* (CaV1.3); *Cacna1f* (CaV1.4); *Cacna1a* (CaV2.1); *Cacna1b* (CaV2.2); *Cacna1e* (CaV2.3); *Cacna1g* (CaV3.1); *Cacna1h* (CaV3.2); *Cacna1i* (CaV3.3). (C) Pearson correlation matrix generated for the qPCR expression profiles of the 11 CaV genes as indicated in (B) for the epididymal fat pads of 13 different male rats. Significant correlation (*P* < 0.05) was seen for all qPCR data sets indicative of reproducibility between animals. R values are coded as shown. (D) RNA-seq data of CaV alpha_1_ subunits expressed as fragments per kilobase of transcript per million mapped reads (FPKM). Ctrl, rat brain; Vis, epididymal; Subc, inguinal subcutaneous fat; *Cacna1s* (CaV1.1); *Cacna1c* (CaV1.2); *Cacna1d* (CaV1.3); *Cacna1f* (CaV1.4); *Cacna1a* (CaV2.1); *Cacna1b* (CaV2.2); *Cacna1e* (CaV2.3); *Cacna1g* (CaV3.1); *Cacna1h* (CaV3.2); *Cacna1i* (CaV3.3). Each point is data from the epididymal adipocytes of a different animal. (E) Linear relationship between the relative expression of the 8 highest expressed CaV genes determined by qPCR with FPKM (>0.02) from RNA-seq. Data are means ± s.d. Line is a best fit with linear regression with a slope of 0.46 (0.16–0.77, 95% C.I.; *P* = 0.0002). (F) RNA-seq data in FPKM of CaV accessory subunits. α_2_δ subunits: *Cacna2d1,Cacna2d2*, *Cacna2d3*, and *Cacna2d4*; β subunits: *Cacnb1*, *Cacnb2*, *Cacnb3*, and *Cacnb4*; γ subunits: *Cacng*4 and *Cacng*7. Each data point is from a different rat. For RNA-seq only data with a geometric mean of >0.1 are shown.

qPCR (Fig. 1A and B) confirm CaV1.2 (*Cacna1c*) as the predominant CaV expressed in rat epididymal and subcutaneous adipocytes; followed by *CaV1.3 (Cacna1d)* and then *CaV2.1 (Cacna1a), CaV2.3 (Cacna1e),* CaV3.1 (*Cacna1g*), CaV3.2 (*Cacna1h*) and CaV3.3 (*Cacna1i*) at similar amounts, with little to no expression of CaV1.1 (*Cacna1s*), CaV1.4 (*Cacna1f*), or CaV2.3 (*Cacna1b*). qPCR integrity was confirmed by the generation of a correlation matrix for the relationships between the 13 rat qPCr CaV expression profiles; this showed that the profiles were similar for all rats (Fig. 1C). The RNA-seq transcriptome of epididymal adipocytes (Fig. 1D) reports a slightly different expression profile to qPCR with CaV3.1 (*Cacna1g*) and CaV3.2 (*Cacna1h*) expressed to a similar level as CaV1.2 (*Cacna1c*). The expression levels of the CaV transcripts from the RNA-seq FPKM data were, however, well correlated (*R* = 0.6, *P* = 0.0002; Pearson) with those from qPCR (Fig. 1E). Figure 1F shows that rat epididymal adipocytes express all known α_2_δ and β accessory subunits, but only *Cacng*4 and *Cacng*7 γ subunits.

Given the relevance of the L-type CaV1.2 and CaV1.3, to adipocyte function (Fedorenko *et al.* 2020) and that of T-type CaV3.1 to adipocyte development (Uebele *et al.* 2009, Oguri *et al.* 2010) we specifically investigated the distribution of these three CaV isoforms in other fat depots and explored how these varied with age and sex.

**Figure 2:**
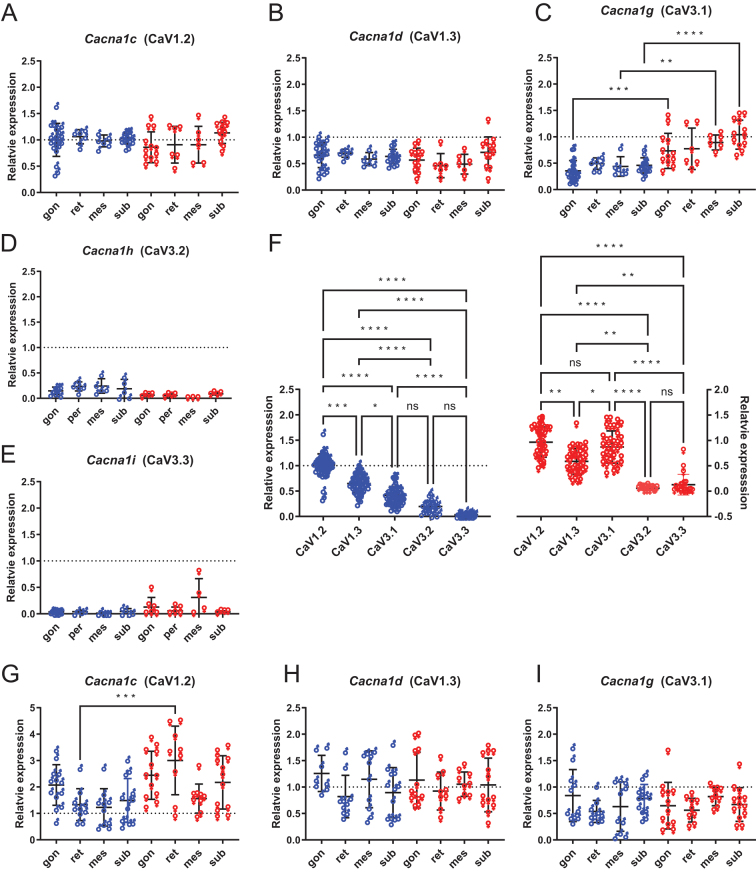
*Cacna1g* genes show differential expression between male and female fat depots of adult rat. Expression of five CaV genes: *Cacna1c* (A, G), *Cacna1d* (B, H), *Cacna1g* (C, I),* Cacna1h* (D), and *Cacna1i* (E) in different fat depots for males (♂) and females (♀) of adult rats age > 140 days postpartum (A–F) and juvenile rats age 14–30 days postpartum (G–I). (F) Relative expression of the genes indicated pooled from the four different depots for males (♂) and females (♀) of adult rats age > 140 days postpartum. gon, gonadal fat, epididymal for males or periovarian for females; ret, retroperitoneal fat; mes, mesenteric fat; sub, IWAT. Data are normalized to mRNA expression of CaV1.2 in male epididymal adipocytes. Statistical significance is by one-way ANOVA, with Sidak multiple comparison test. For D, E, and F, statistical significance is by Kruskal–Wallis with Dunn’s multiple comparison test. Each point represents a sample from a different animal; for adults *n* = 6–20, juveniles *n* = 8–13. A full colour version of this figure is available at https://doi.org/10.1530/JME-23-0108.

For gonadal, retroperitoneal, mesenteric, and IWAT depots (Fig. 2A, B, C, D, and E) of adults of a given sex there were no significant differences in the levels of transcript for *Cacna1c* (CaV1.2),* Cacna1d* (CaV1.3), *Cacna1g* (CaV3.1)*, Cacna1h* (CaV3.2), or *Cacna1i* (CaV3.3) (one-way ANOVA, Kruskal–Wallis). In adults, the expression levels of *Cacna1c,Cacna1d, Cacna1h*,and *Cacna1i* also did not differ between sexes (Fig. 2A, B, D, and E); however, the expression of* Cacna1g* (CaV3.1) was significantly greater in females than in males for gonadal, mesenteric, and IWAT but not retroperitoneal depots (Fig. 2C; one-way ANOVA). The expression levels of these five genes from the different depots data was pooled from adults for either sex (Fig. 2F). For male adult rats the rank order of CaV gene expression was CaV1.2 > CaV1.3 > CaV3.1 > CaV3.2 = CaV3.3, whereas in female adult rats the rank order of CaV gene expression was different: CaV3.1 ≥ CaV1.2 > CaV1.3 > CaV3.2 = CaV3.3 – a variation due to the heightened expression of CaV3.1 (*Cacna1g*). A different picture for gene expression was observed in juvenile rats (p15-30) (Fig. 2G, H, and I), where, with the exception for *Cacna1c* in juvenile rat retroperitoneal, there was no significant difference (one-way ANOVA) in the expression of *Cacna1c*,* Cacna1d*, or *Cacna1g* between either depot or sex.

**Figure 3:**
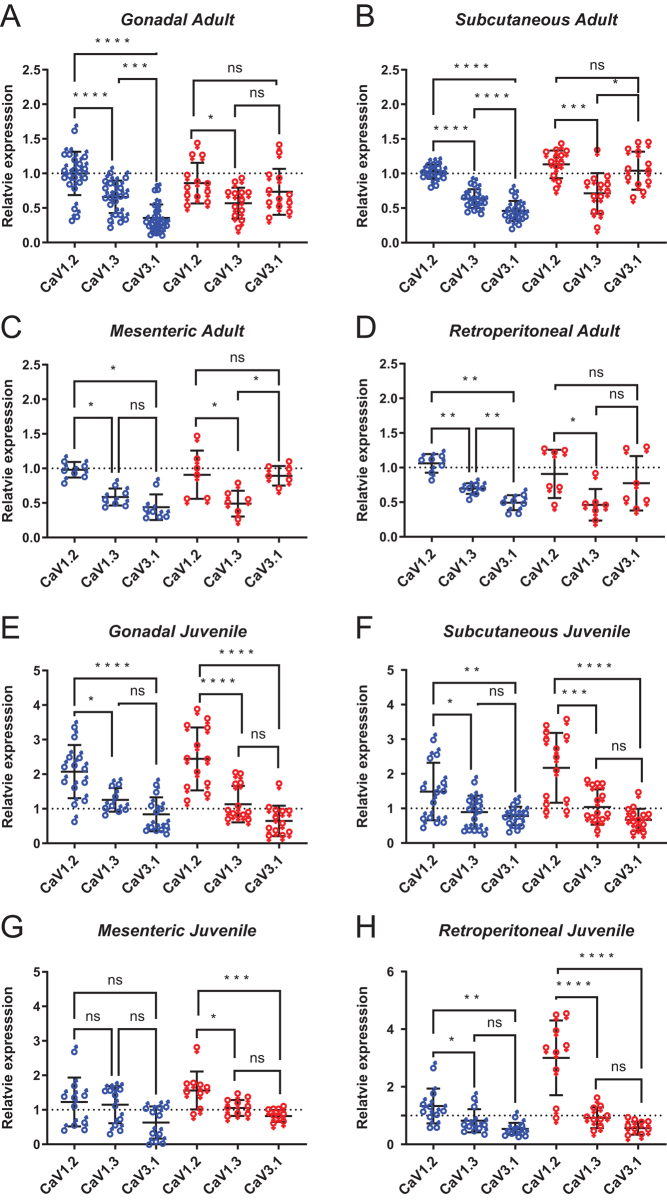
Cacna1 genes show similar rank order of expression in fat depots of juveniles and adult male but not adult female rat. Expression of the three CaV genes: *Cacna1c* (CaV1.2), *Cacna1d* (CaV1.3), and *Cacna1g* (CaV3.1) in different fat depots for males (♂) and females (♀) of adult rats (age > 140 days postpartum) (A, B, C, D) and juvenile rats (age 14–30 days postpartum) (E, F, G, H) as indicated. Data are normalized to mRNA expression of CaV1.2 in male epididymal adipocytes. Due to the low expression of CaV3.2 and CaV3.3 these are omitted for clarity. Statistical significance is by one-way ANOVA, with Tukey multiple comparison test. Each point represents a sample from a different animal; for adults *n* = 6–20, juveniles *n* = 8–13. A full colour version of this figure is available at https://doi.org/10.1530/JME-23-0108.

Figure 3 shows the relative expression levels of *Cacna1c*,* Cacna1d*, and *Cacna1g* compared within a depot type. For adult rat depots the rank order of CaV gene expression was CaV1.2 > CaV1.3 > CaV3.1 for male rats, whereas in females the rank order was different: CaV3.1 ≥ CaV1.2 > CaV1.3, as shown in Fig. 2F. The rank order of expression for juveniles rats of both sexes, other than for male juvenile mesenteric possibly due to a type II statistical error, were the same: CaV1.2 > CaV1.3 > CaV3.1 (CaV3.2 and CaV3.3 not detected).

**Figure 4:**
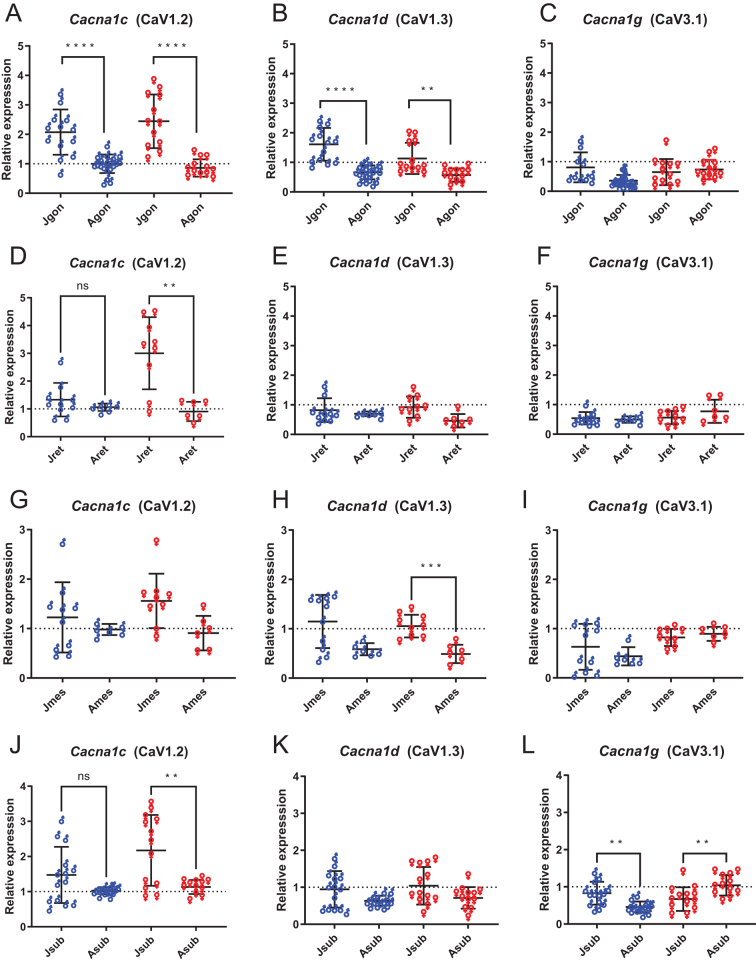
Cacna1 genes show changes in expression with age. Expression of the three CaV genes: *Cacna1c* (A, D, G, J), *Cacna1d* (B, E, H, K), and *Cacna1g* (C, F, I, L) in different fat depots for males (**♂**) and females (**♀**) from juvenile rats (age 14–30 days postpartum) (J prefix) and adult rats (age > 140 days postpartum) (A prefix). gon, gonadal fat (A, B, C), epididymal for males or periovarian for females; ret, retroperitoneal fat (D, E, F); mes, mesenteric fat (G, H, I); sub, IWAT (J, K, L). Data are normalized to mRNA expression of CaV1.2 from male epididymal adipocytes. Statistical significance is by Welch’s ANOVA, with Dunnett’s T3 comparison test with significance set at *P* of < 0.01 to account for statistical comparisons performed on this data in Fig 2. Each point represents a sample from a different animal, for adults *n* = 6–20, juveniles *n* = 8–13. A full colour version of this figure is available at https://doi.org/10.1530/JME-23-0108.

Since the relative expression for *Cacna1c*, *1d*, and *1g* in adult rats (Fig. 2A, B, and C) appeared to be smaller than that found in juveniles (Fig. 2G, H, and I) we explored the effect of age on gene expression. Figure 4 shows that the expression of *Cacna1c* (CaV1.2) differentially changed with sex post puberty, with decreases seen in gonadal, retroperitoneal and IWAT depots for female rats (Fig. 4A, D, and J), whereas for males a decrease was only seen in gonadal depots (Fig. 4A). *Cacna1d* (CaV1.3) also changed expression with age, but this phenomenon was dependent on both depot and sex; with decreases seen in gonadal for both sexes and for female rats only in mesenteric depots (Fig. 4B and H). As for *Cacna1g* (CaV3.1), its expression changed with age only in IWAT depots (Fig. 4L), with a ~2-fold decrease in males and a ~2-fold increase in females IWAT (Fig. 4L). In summary, with exception of *Cacna1g* (CaV3.1) in inguinal (mammary) depots in females which increased, the three CaVs isoforms either did not change expression or were down-regulated post puberty. Interestingly the coefficient of variation decreased by 14 ± 18% (*P* < 0.001 paired *t*-test) with post puberty, data indicative of greater transcript variation in the juvenile depots.

**Figure 5:**
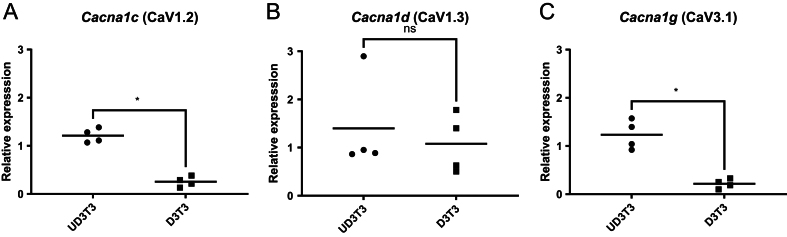
Expression of *Cacna1* genes in 3T3-L1 cells is affected by differentiation. Expression of the three CaV genes: *Cacna1c* (A), *Cacna1d* (B), and *Cacna1g* (C) in undifferentiated (UD3T3) and differentiated (D3T3) 3T3-L1 cells. Data are normalized to mRNA expression of CaV1.2 in epididymal fat. Statistical significance is by Mann–Whitney. Each point represents a sample from a different passage number (*n* = 4). There was no significant change in the coefficient of variation (paired *t*-test).

A commonly used adipocyte cell-line model are 3T3-L1 (mouse fibroblast) cells (Oguri *et al.* 2010, Bentley *et al.* 2014). We compared the gene expression for *Cacna1c*, *Cacna1d*, and *Cacna1g* in undifferentiated and differentiated 3T3-L1 cell lines (Fig. 5A, B, and C). *Cacna1c* (CaV1.2) and *Cacna1g* (CaV3.1) had significantly greater expression in undifferentiated 3T3-L1 pre-adipocytes than when differentiated into adipocytes (Fig. 5A and C). *Cacna1d* (CaV1.3) expression did not differ between differentiated and differentiated 3T3-L1 cells (Fig. 5B).

**Figure 6:**
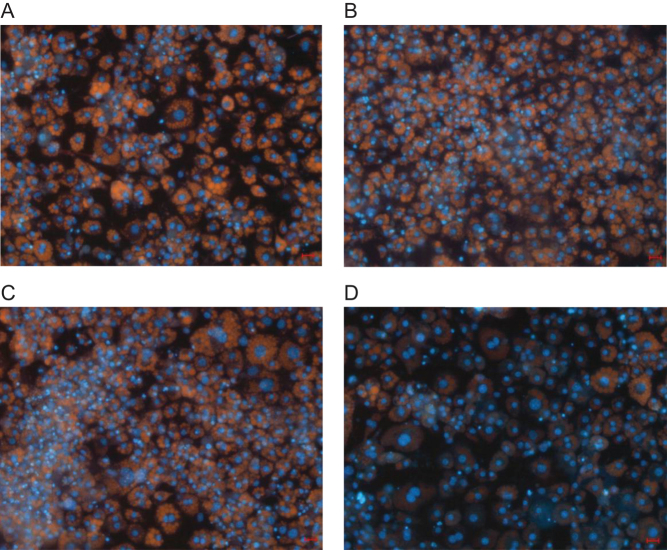
Images of differentiated 3t3-L1 adipocytes stained with Hoechst 33342 and Nile Red in the presence of CaV blockers. (A) Control, (B) 10 µM mibefradil, (C) 50 µM TTA-A2, (D) 10 nM calciseptine. Cells were incubated for 8 days in drugs and vehicle control. Scale bar 10 µM.

**Figure 7:**
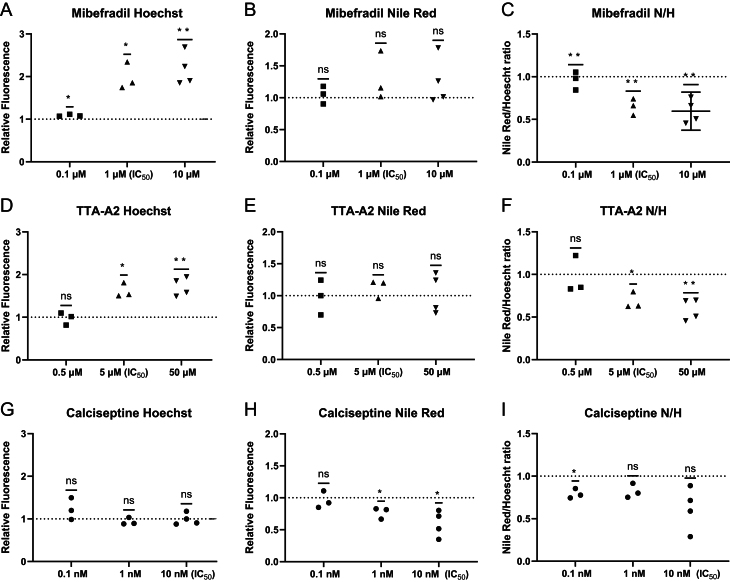
Inhibition of CaV3.1 promotes pre-adipocyte proliferation but does not affect adipocyte differentiation. Effect of CaV blockers on cell proliferation measured by staining with Hoechst, differentiation measured by staining with Nile Red and adipogenesis by the ratio of Nile Red to Hoechst staining (N/H). Fluorescence values for drugs are normalised (relative fluorescence) to their respective vehicle control: DMSO for mibefradil and TTA-A2, water for calciseptine. Statistical significance is by One-sample test. Each point represents a sample from a different passage number (*n* = 3–4).

Give the putative role of CaV3.1 in adipocyte development (Uebele *et al.* 2009, Oguri *et al.* 2010) we investigated the effect of the selective blockers of this channel: mibefradil and TTA-A2 on preadipocyte proliferation and differentiation. Both mibefradil and TTA-A2 at their respective IC_50_ values promoted proliferation, as revealed by Hoechst staining (Figs. 6 and 7). However, although the ability of the cell population to differentiate and store lipid as indicated by their ability to accumulate Nile Red was like control cells (Figs. 6 and 7), the fact the cells were almost twice as dense as control cells suggests differentiation was impaired – an idea supported by the significant decrease in the Nile Red/Hoechst ratios (Figs. 6 and 7). Calciseptine, a selective peptide inhibitor of CaV1.x was without effect on either proliferation or differentiation (Figs. 6 and 7).

## Discussion

Consistent with our previous work (Fedorenko *et al.* 2020) we found the predominant voltage-gated calcium channels genes expressed in rat epididymal fat are *Cacna1c* (CaV1.2), *Cacna1d* (CaV1.3), and *Cacna1g* (CaV3.1). We now show that CaV1.2 and CaV1.3 gene expression does not differ between gonadal, retroperitoneal, mesenteric, and inguinal subcutaneous (IWAT) fat depots for juvenile or adult rats of either sex. Moreover, except for CaV1.2 in juvenile retroperitoneal fat, no difference between sexes was observed for the expression levels for CaV1.2 and CaV1.3 for either juvenile or adult rats. In contrast, in female compared to male adults, but not juveniles, CaV3.1 had a greater expression in adipocytes from gonadal, mesenteric and IWAT, depots . Although CaV3.1 expression did not change with puberty in retroperitoneal, mesenteric, and gonadal depots for either sex, it did change in IWAT depots: whereas CaV3.1 decreased in males it increased with puberty in females.

Transcripts for CaV3.2 and CaV3.3 were often undetectable. since differentiated 3T3-L1s adipocytes do not demonstrate CaV3.2 and CaV3.3 transcripts (Zhang *et al.* 2017*b*) an adipocyte origin is doubted for these two transcripts. Transcripts for CaV3.2 and CaV3.3, but not CaV3.1, are found in macrophages, a cell type which contaminates our adipocyte preparations as indicated by the presence of their specific marker genes: Tyrobp and Adgre1 (Ying *et al.* 2017). The fact that transcriptomes of human and mouse immune T cells show CaV2.1, CaV3.2, and CaV3.3 in human, and CaV2.1 and CaV3.2 in mouse but with little evidence of CaV3.1, suggests that the CaV3.1 observed in the present study is solely attributed to white fat adipocytes (Heng *et al.* 2008, Erdogmus *et al.* 2022).

Our data suggest that CaV3.1 has a crucial role in female adult adipose tissue that relates to sexual dimorphism in fat deposition and function (Newell-Fugate 2017). Indeed, CaV3.1 is established to be upregulated in the hypothalamus and pituitary of rodents by a process that involves the female sex hormone oestrogen (Qiu *et al.* 2006, Zhang *et al.* 2009); however, the signalling cascade has yet to identified.

With the onset of puberty a two-fold increase in the serum oestrogen oestradiol (E2) ensues in female (P30–P40 rats) (Parker & Mahesh 1976) but not in male rats (Bell 2018). The actions of oestrogens in adipose are depot dependent: decreasing visceral but not subcutaneous adiposity (Cooke *et al.* 2001, D’Eon *et al.* 2005, Bjune *et al.* 2022); this idea is supported by the observation that global nuclear oestrogen receptor ESR1 (ERα) knockout animals become susceptible to obesity via visceral fat gain (Cooke *et al.* 2001, Foryst-ludwig *et al.* 2008, Winn *et al.* 2023) – a finding consistent with humans where ESR1 expression transcript is negatively correlated with female waist-to-hip ratio indicative of subcutaneous weight gain (Ahmed *et al.* 2022). With regard to sexual dimorphism, ESR1 protein expression is found to be larger in inguinal (mammary) subcutaneous adipose from post-pubescent female mice and pigs compared to their age-matched male counterparts (Winn *et al.* 2023), thus mirroring the *Cacna1g* transcript expression profile we found for adult rats. This finding supports the idea that oestrogen may promote/maintain *Cacna1g* transcription in adipocytes, like that established in rodent brain (Qiu *et al.* 2006, Zhang *et al.* 2009), or alternatively, ESR1 and CaV3.1 are permissive partners for IWAT development.

Given the permissive role extracellular Ca^2+^ influx plays in sympathetic mediated lipolysis (Izawa *et al.* 1983), an increased functional expression of CaV3.1 in post-pubescent female fat tissue may enhance this process like that observed when CaV1.x activity is augmented by dihydropyridine agonists (Fedorenko *et al.* 2020). In an additional study (Supplementary Information, see section on supplementary materials given at the end of this article) we observed no difference in basal [Ca^2+^]_i_ or cell diameter in adipocytes isolated from male and female adult mouse IWAT, thus suggesting that CaV3.1 is not constitutively active within these depots and does not contribute to basal [Ca^2+^]_i_. However, in order to affect adipogeneses it must be regulated by extrinsic factors necessary to promote adipocyte differentiation, for example oestrogen (Uebele *et al.* 2009, Oguri *et al.* 2010). The decrease in the coefficient of variation post puberty is something that was not observed using the cell line, thus suggesting that pre-pubescent primary tissue contains a range of developmental states that are lost on adulthood.

Since adipogenic oestrogen is critical to the development of the murine female reproductive system and function (Wang *et al.* 2017), the increased CaV3.1 expression we detect in murine ovarian fat relative to adult males may well also relate to adipogenesis and the subsequent provision by these adipocytes of suitable local paracrine interactions to develop and maintain the rodent female reproductive tract (Gui *et al.* 2004, Reverchon *et al.* 2014). WFA have a key role in the synthesis and storage of oestrogens (Cannady *et al.* 2000). In particular they express aromatase which catalyses the conversion of adrenal androgens, such as testosterone and androstenedione, to the aromatic oestrogens (oestradiol (E2) and oestrone (E1)) (Mair *et al.* 2020). Indeed removal of periovarian fat in mice leads to a several-fold decrease in circulating oestrogen levels, irregular oestrus, impaired folliculogenesis, and infertility (D’Eon *et al.* 2005, Wang *et al.* 2017) However, since humans do not appear to have gonadal fat (Chusyd *et al.* 2016, Börgeson *et al.* 2022) these conclusions from rats do not translate to man. Moreover, omental fat is the major visceral depot in man whereas in rodents this is gonadal, with neither depot possessed by the other species. In man, omental fat subserves an immunomodulatory role where weight gain is by adipocyte hypertrophy, whereas in rodents gonadal fat serves an endocrine and lipid storage role, and weight gain is by adipocyte hyperplasia (Börgeson *et al.* 2022); differences that contrast the physiology of inter species visceral fat and precludes translation inference.

Conversely and importantly, murine IWAT contains the fourth and fifth mammary glands (Grove *et al.* 2010, Chusyd *et al.* 2016) which humans do possess; with WFA established to be critical in mammary ductal formation and functional plasticity for both mouse and man (Kothari *et al.* 2020, Colleluori *et al.* 2021). Consequently, the changes in CaV3.1 expression profiles we observed in IWAT may relate to the established association between the paracrine action of adipocytes and mammogenesis (Colleluori *et al.* 2021). CaV31.1 is unlikely to relate to subcutaneous weight gain in rodents and humans, since this predominantly occurs by a process of hypertrophy and not by hyperplasia (Börgeson *et al.* 2022).

CaV3.1 blockers such as pimozide and mibefradil are shown to be antiproliferative in adipose models of breast cancer (Bertolesi *et al.* 2002), an observation we now confirm for 3T3-L1 adipogenesis. Indeed, antagonists of T-type VGCCs are proposed to be a potential treatment for breast cancer (Bhargava & Saha 2019). The abilities of *in vivo* administration of TTA-A2, a CaV3.1 selective antagonist, as well as that of genetic knockout of *Cacna1g* (CaV3.1) to both inhibit of high-fat weight gain in mice (Uebele *et al.* 2009) emphasize the importance of CaV3.1 in adipogenesis; our observations with the ability of TT-A2 and mibefradil, selective blockers of CaV3.1, but not that of CaV1.x, calciseptine, to inhibit adipogenesis of 3T3-L1 cells are consistent with this idea. However, at 10 µM, mibefradil can mobilise intracellular Ca^2+^ stores (Souza Bomfim *et al.* 2021), so interpretation of its effects should be treated with caution.

With the retroperitoneal and mesenteric depots, it is difficult to account for the decreases in expression seen for CaV1.2 and CaV1.3 in these depots post puberty, especially since they are considered to have negligible roles in systemic metabolism (Börgeson *et al.* 2022).

Unlike primary adipocytes, gene expression in undifferentiated 3T3-L1 pre-adipocytes was similar for all three channel isoforms: CaV3.1 ~ CaV1.2 ~ CaV1.3, where the CaV3.1 and CaV1.2 transcript levels decreased with differentiation – an observation we observed post puberty except for CaV3.1 in female depots associated with secondary sexual characteristics. Our results for CaV3.1 expression in 3T3-L1 cells confirm those previously reported in this cell line (Uebele *et al.* 2009, Oguri *et al.* 2010). The decrease in CaV3.1 expression we observed after differentiation may be associated with a subsequent redundancy of functional protein; as confirmed with the loss of CaV3.1 protein in Western blots with 3T3-L1 on differentiation (Oguri *et al.* 2010) (supplementary information 2). Although our 3T3-L1 data support the involvement of CaV3.1 in the differentiation of pre-adipocytes into mature adipocytes, we draw the contrary conclusion for others in regard to proliferation (Oguri *et al.* 2010). Although we observed that the pharmaceutical inhibition of CaV3.1 decreased differentiation, where adipogenesis is associated with enhanced Ca^2+^ influx (Zhang *et al.* 2018), inhibition of CaV3.1 actually augmented proliferation. The latter phenomenon is explained by our use of a longer, 10-day compared to a 24-h drug incubation(Oguri *et al.* 2010), a procedure necessary to encompass the cell cycle length of 30–40 h.

With regard to the sex-dependent differences we observed for CaV expression, this may be a case of differential gene regulation by gonadal hormones such as oestrogen (D’Eon *et al.* 2005, Qiu *et al.* 2006) or alternatively it may relate to the autosomal genome (Newell-Fugate 2017, Chang *et al.* 2018).

RNA-seq revealed expression of the membrane G protein-coupled oestrogen receptor gene transcript, GEPR1 (GRP30), and the nuclear oestrogen receptor ESR1 (ERα), of which ESR1 had the greatest expression, a transcript not found in immune cells and so is likely to arise solely from adipocytes; ESR1 and GPER1 were expressed with median FPKMs of 15.3 and 0.37, respectively – thus supporting the existence of oestrogenic signal cascades in primary white fat adipocytes.

Genes like *Cacna1g* (CaV3.1) and *Cacna1c* (CaV1.2) are sexually dimorphic at both the transcriptomic and functional level in murine tissues such as brain (Qiu *et al.* 2006, Zhang *et al.* 2009) and heart (Sims *et al.* 2008), and as we now report, in white fat adipocytes too.

### Physiological relevance

Mature primary adipocytes and differentiated 3T3-L1s have a plasma membrane potential that ranges between −50 to −15 mV (Bentley *et al.* 2014): voltages sufficiently depolarised to constitutively activate both CaV1.2 and Ca3.1 and elicit a sustained window Ca^2+^ influx (Crunelli *et al.* 2005, Fedorenko *et al.* 2020, Akaniro-Ejim & Smith 2021). Although, at these membrane potentials, CaV3.1, but not CaV1.2, may be expected to undergo voltage-dependent inactivation, this may not occur, since in adipocytes its voltage dependence of inactivation is sufficiently shallow to prevent complete inactivation (Oguri *et al.* 2010) especially in those cells with a more negative Vm. Consequently, functional activity of CaV3.1 would permit constitutive Ca^2+^ influx (Crunelli *et al.* 2005, Fedorenko *et al.* 2020) and impart sensitivity to CaV3.1 blockers as we observed in the present study. Since adipocytes are electrically inactive (Bentley *et al.* 2014), CaVs and Ca^2+^ influx is not modulated by changes in membrane potential but by other means; for example, growth hormone activates CaV1.2 and promotes Ca^2+^ influx in mouse primary adipocytes (Akaniro-Ejim & Smith 2021). Our present data suggest that CaV3.1 maybe upregulated through either gene expression, possibly via oestrogen as seen in neuronal tissues (Qiu *et al.* 2006, Newell-Fugate 2017) or/and by channel phosphorylation by the cyclin-dependent kinase, CDK5, a process observed for CaV3.1 in neurons and found critical for their differentiation (Calderón-Rivera *et al.* 2015). In fact our rat RNA-seq show expression of CDK5 transcripts, as do differentiated 3T3-L1s (Zhang *et al.* 2017*a*) with FPKM geometric means of 10 and 13, respectively; moreover, both types of cells express CKD5 protein (Lalioti *et al.* 2009). The fact that CDK5 mediates phosphorylation of proteins involved in 3T3-L1 differentiation (Lalioti *et al.* 2009) highlights this kinase as a realistic candidate to activate CaV3.1 during adipogenesis.

Together, our data suggest that the elevated expression of CaV3.1 in females is involved in depots that have a dynamic growth profile such as murine and human mammary glands where changes occur with pregnancy, lactation, and involution, as well as oestrus in rodents. However, to do so, we suggest that extrinsic factors such as oestrogen must modulate the functional activity of CaV3.1 to affect its cellular actions. That CaV3.1 is an established clinical drug target to treat human breast cancer and that CaV3.1 antagonists inhibit weight gain in mice suggest that modulation of this CaV may be beneficial in the regulation of secondary sexual characteristic functions.

## Supplementary Materials

Supplementary Material

## Declaration of interest

The authors declare that there is no conflict of interest that could be perceived as prejudicing the impartiality of the study reported.

## Funding

This work did not receive any specific grant from any funding agency in the public, commercial, or not-for-profit sector.
