# Supplementary material for: Sex and age affect depot expression of Ca2+ channels in rat white fat adipocytes
Source: J Mol Endocrinol. 2024 Feb 28;72(4):e230108. doi: 10.1530/JME-23-0108 (PMC10959010; doi:10.1530/JME-23-0108)
Supplement: Supplementary Material [file supplementary_material.pdf]

Supplementary Information

1. Sex does not affect cell diameter or basal intracellular Ca^{2+} concentration.

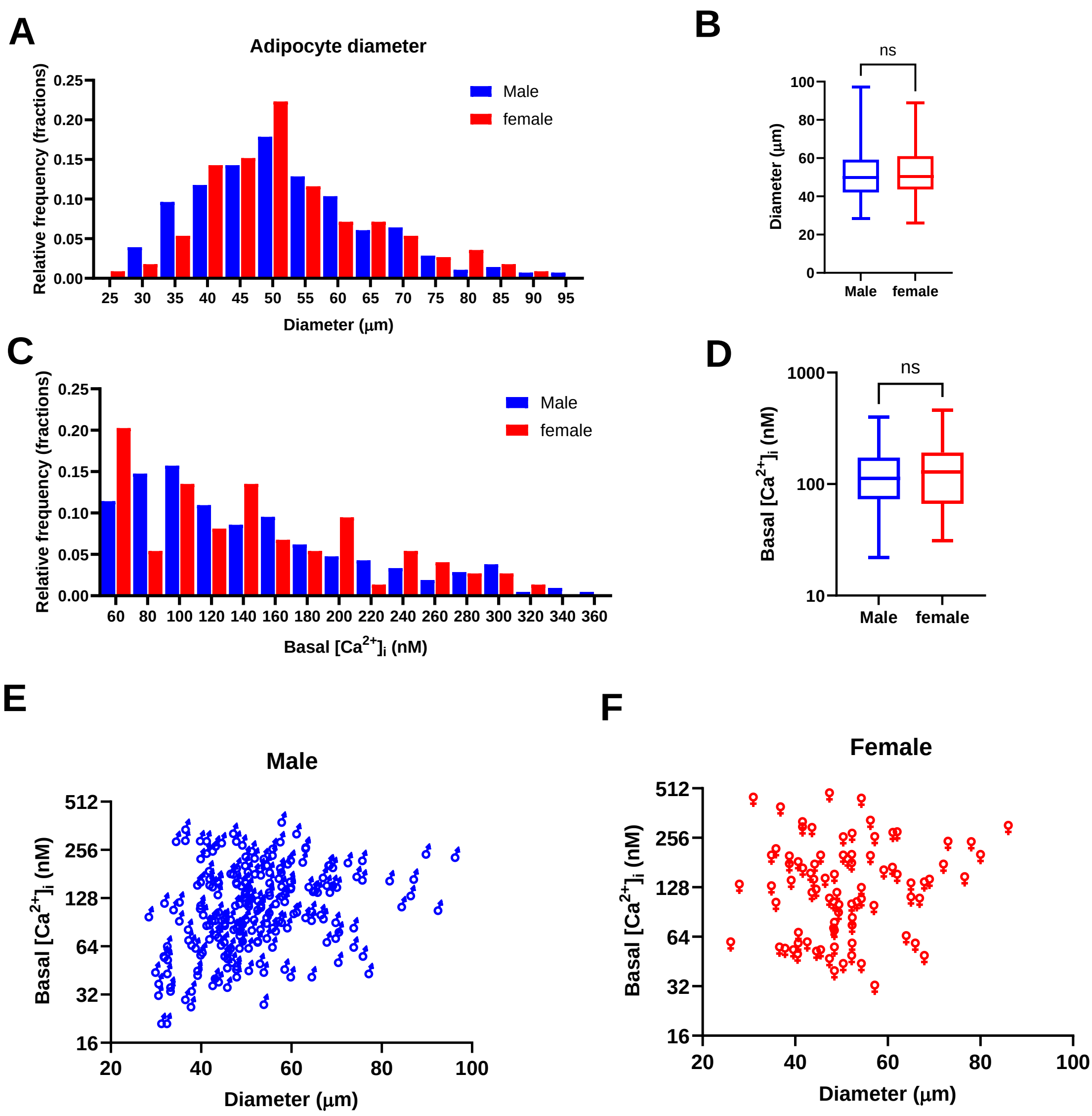

Frequency distributions for cell diameter (A) and basal $[Ca^{2+}]_i$ (C) of primary male and female adult mouse inguinal adipocytes No difference was seen in either diameter (B) (50.2±1.3 vs 51.2±1.3 μm, p=0.51), or basal $[Ca^{2+}]_i$ (Fig. 1D) (110.6±1.8 vs 120.0±1.9 nM, p=0.1) between male (n=240-280, A= 16) and female (n =86-112, A=4) subcutaneous inguinal adipocytes. Male (E) (r=0.29, p<0.001), but not female (F) (r=0.076, p=0.48), adipocytes show a significant but weak (r^2=0.08) correlation of $[Ca^{2+}]_I$ with cell diameter.

Methods. Primary adipocytes were isolated by collagenase digestion from inguinal fat pads (4th and 5th mammary fat pad) of post-pubescent (P25-35) virgin CD-1 mice. Mice were purchased at 33-31g weight (♂ >P35; ♀ >P56) (Charles rivers) housed as single sex cohorts and used within 2 weeks of arrival. Cytosolic Ca^{2+} concentration, $[Ca^{2+}]_i$, was measured by epifluorescent videomicroscopy with the fluophore, Fluo4 and calibrated with 0.05% vol/vol TX-100 as previously described (Fedorenko *et al.*, 2020; Akaniro-Ejim and Smith, 2021). Data are described as geometric means with SD factor; data presented for male followed by female (n = number of cells, A = number of animals). Comparisons are made with Mann-Whitney tests, corelation with Spearman r. Boxplots are min-max. Statistical significance is defined as P<0.05.

2. CaV3.1 protein expression decreases with adipocyte differentiation.

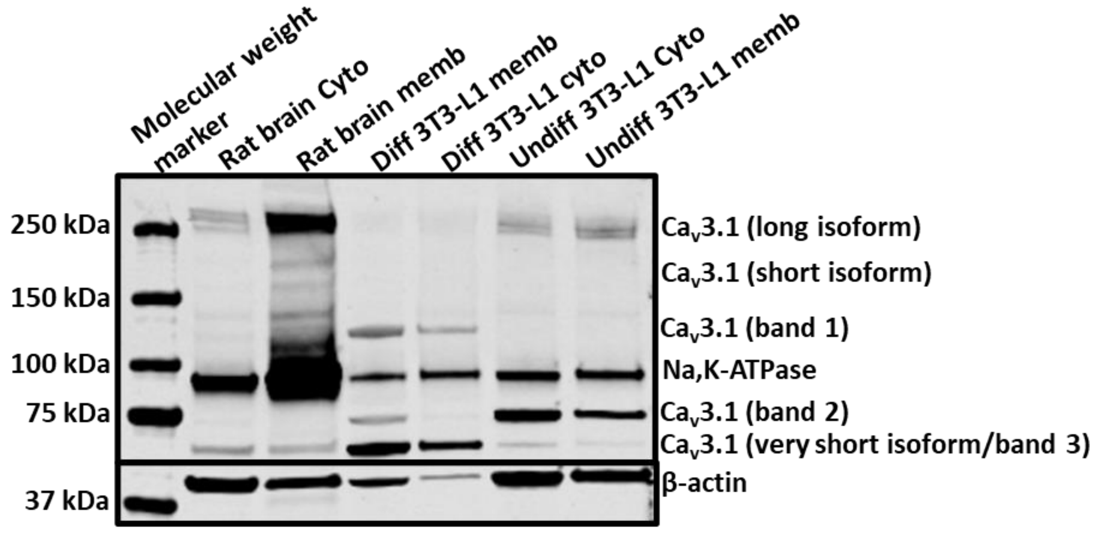

Representative western blot of $Ca_V3.1$ and Na, K-ATPase protein expression in cytosolic (Cyto) and membrane (memb) fractions of Rat brain, differentiated 3T3-L1 adipocytes (Diff) and undifferentiated 3T3-L1 cells (Undiff). The detection of the full length $Ca_V3.1$ α_1 subunit of ~265kDa in 3T3-L1 pre-adipocytes in this study is consistent with previous observations, with its expression levels higher in the undifferentiated adipocytes. The presence of different channel isoforms could arise due to alternative splicing of these channels, alternative epitopes, or proteolysis.

Methods. Western blots were performed as previously described for this cell type (Fedorenko *et al.* 2020). Proteins were detected by overnight incubation at 4 °C with primary antibodies anti CaV3.1 (Alomone ACC-021) at a 1:500 dilution, anti Na,K-ATPase at 1:20,000 (Abcam ab76020)and anti β-actin (Sigma A1978) at 1:5000 dilution. Membranes were washed for 6 x 5 minutes with TBST and incubated for 1 hour with a 1:10,000 dilution of infra-red dye labelled secondary antibodies; IRDye 800 CW goat anti-rabbit IgG (Li-COR™ 926-32211) and IRDye 680 CW goat anti-mouse IgG (Li-COR™ 926-68070). All antibody dilutions were made in 5 % milk in TBST.
